# Beneficial effects of Cyclosporine A in combination with Nortriptyline on germ cell-specific apoptosis, oxidative stress and epididymal sperm qualities following testicular ischemia/reperfusion in rats: a comparative study

**DOI:** 10.1186/s40360-022-00601-6

**Published:** 2022-08-05

**Authors:** Iraj Yazdani, Raheleh Majdani, Morteza Ghasemnejad-berenji, Ahmad Reza Dehpour

**Affiliations:** 1 Department of Pharmacology, School of Medicine, Tehran University of Medical Sciences, P.O. Box 13145-784, Tehran, Iran; 2 Department of Cellular and Molecular Biology, Faculty of Basic Science, University of Maragheh, Maragheh, Iran; 3 Department of Pharmacology and Toxicology, School of Pharmacy, Urmia University of Medical Sciences, Urmia, 5715799313 Iran

**Keywords:** Apoptosis, Cyclosporine A, Nortriptyline, Oxidative stress, Sperm, Testicular T/D

## Abstract

**Background:**

Testicular torsion is a pathological condition which needs emergency surgical intervention. However, after surgical reperfusion, oxidative stress factors cause to germ cell apoptosis. The study was planned to evaluate the efficacy of simultaneous use of Cyclosporine A (CsA) and Nortriptyline (Nort) to repair testicular damages in an experimental torsion/detorsion (T/D) rat model.

**Methods:**

Male rats (*n* = 112) were allocated into 7 groups 16 each in; (Group 1); Control group, (Group 2); T/D group, (Group 3–4); CsA 1 and 5 mg/kg, (Group 5–6); Nort 2 and 10 mg/kg and (Group 7); concurrent group, CsA (1 mg/kg) + Nort (2 mg/kg). Right uni-lateral torsion was inducted by twisting testis 720 degrees in the clockwise direction for 1 h. For short-term and mid-term studies, lipid peroxidation, antioxidant enzyme activities, caspase-3 level, histopathological changes and germ cell apoptosis were evaluated. Moreover, in long-term investigation, semen analysis was performed.

**Results:**

After T/D induction, testis abnormalities both functional and structural were appeared. Pre- and post-treatment with CsA and Nort, separately, reduced MDA and caspase-3 levels, normalized antioxidant levels, ameliorate tissue injury and improved sperm criteria.

**Conclusion:**

The antioxidant and anti-apoptotic characteristics of CsA and Nort and their protective effects have been shown in our study. Concurrent administration of CsA and Nort in selected low-dose indicated a significant positive effect as compared to the individual drug interventions on the reversal of T/D induced oxidative stress in short-term, apoptosis, and histologic changes in mid-term, as well as semen criteria in the long-term appraisal.

## Introduction

Testicular torsion a traumatic state in men urologic system with an annual incidence rate of 1 in 158 which needs the emergency surgical intervention to avoid loss of testis function and infertility consequences [1]. Although the exact reasons remain unclear, detrimental events and mediators in testicular T/D are similar to ischemia/reperfusion (I/R) mechanisms; ischemia in torsion followed by reperfusion in detorsion [2]. As a first step, immediate restoration of oxygen and glucose by blood flow is necessary for deprived tissue. Despite the re-oxygenation, free radicals produced during reperfusion can lead to generation of excessive intracellular toxic reactive oxygen species (ROS) and indices of inflammation which can deteriorate trauma even with successful surgical repair [3, 4]. Oxygen free radicals oxidize membrane lipids and proteins which disrupts membrane integrity may play an important role in leydig cell abnormality and disruption in spermatogenesis [5]. Albite multifactorial mechanisms mediate this condition, the primary insult inception in I/R is releasing of immense intracellular calcium (Ca^2+^) ions and loss of mitochondrial transmembrane potential (ΔΨm) in ischemia phase and generation of ROS with swelling of mitochondrial inner membrane via pressure of accumulated H_2_O solutes in reperfusion phase which exacerbate dysregulation of respiratory chain in mitochondria and predispose cell to oxidative stress with reactive free radicals by opening of mitochondrial permeability transition pore (mPTP) [6–8]. mPTP channel is a non-specific channel in mitochondrial inner membrane and in I/R conditions its opening allows to accumulated H_2_O molecules to pass through the mitochondrial membrane [6]. Mentioned pathologic processes will finish by cell death due to activation of caspase pathways through releasing of pro- apoptotic factors such as cytochrome *c* and apoptotic induced factor (AIF) from mitochondrial inter-membrane space resulted from opening of mPTP [6, 9–11]. In this regard inhibiting this pathologic process by mPTP inhibitors could ameliorate I/R induced injuries [12]. Pervious investigations have clarified that cyclosporin A (CsA) is able to inhibit mPTP opening in I/R [2, 3]. Hence, the cytoprotective effects of CsA following I/R in various experimental models such as, myocardial infarction, liver I/R, testicular T/D and brain ischemia have been reported previously [13–18]. On the other hand, nortriptyline (Nort), a well-tolerated second-generation tricyclic antidepressant with a wide therapeutic index, is described as a strong mPTP blocker and its main mechanism in treatment of injuries caused by I/R may be relate to mPTP opening [19, 20]. Numerous *in vivo* and *in vitro* researches have showed the anti-inflammatory, anti-apoptotic and cytoprotective effects of Nort in brain and testis in I/R condition. These effects rely on its ability to block the activity of inflammatory mediators, including tumor necrosis factor-alpha (TNF-α), interleukin-1 β (IL-1β), IL-6, caspase-3 and the activation of mPTP in I/R injuries [2, 19, 21]. Accordingly, in the present study for the first time the effects of simultaneously treatment with CsA and Nort, two potent mPTP inhibitor agents, on biochemical, histopathological and epididymal semen quality in a rat model of testicular I/R were evaluated. The aim of this study was to clarify whether combined CsA—Nort therapy would be superior to either one alone for protecting the testis against reperfusion injury.

## Material and methods

### Animals and ethics

Totally 112 mature male Wistar rats weighing 220-250 g were supplied from Tehran University of Medical Sciences and were kept in 23 ± 2 °C with free access to food and water. The authors envisaged all standard protocols in accordance with the 1964 Declaration of Helsinki and Tehran University of Medical Sciences Ethics Committee (NO. 92.02.10–66). All methods are reported in accordance with ARRIVE guidelines.

### Drugs and treated groups

**Fig. 1:**
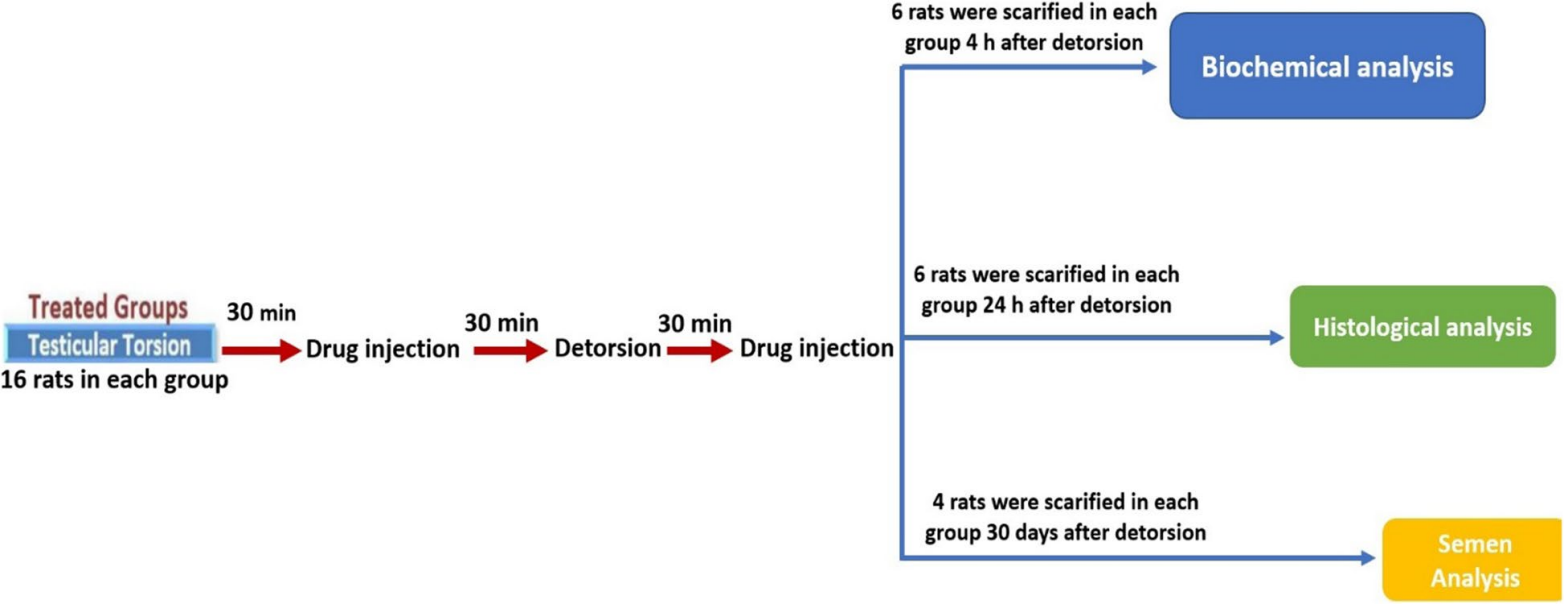
Schematic diagram of the experimental design

Schematic diagram of the experimental design is shown in Fig. 1. CsA (Novartis Pharma, Basel, Switzerland) was dissolved in 0.1% DMSO and injected *i.v*. Nort (Darou Pakhsh Pharmaceutical Co. Tehran, Iran) was dissolved in normal saline 0.9% and administered intraperitoneally. Ketamine HCL (Gedoon Richter Ltd., Budapest, Hungry) and Xylazine (Alfasan Pharmaceutical Co. Netherlands) were used as anesthesia agents. All used compounds were given, freshly. Totally 112 mature male Wistar rats were divided in seven groups (*n* = 16 each). Right testes of six animals in each group were removed 4 h after the beginning of testicular detorsion to rule out the alterations in lipid peroxidation and antioxidant enzymes activities induced by testicular T/D [22, 23].

The procedure was continued for six other animals undergone T/D from each group to assessment the rate of germ cell apoptosis 24 h after reperfusion, which is its maximum peak level [2, 23]. Eventually, right testis of four remaining rats in each group was removed to determine spermatic functions 30 days after treatment [24]. Specific doses of CsA (1and 5 mg/kg) and Nort (2 and 10 mg/kg) were administrated *iv* and *i.p* respectively, 30 and 90 min. after testicular torsion. Indeed, the early dose was given 30 min. before detorsion and the later dose was given 30 min. after detorsion; Experimental groups were arranged as follows (*n* = 16 per group):Control group; served as sham-operated group; only surgical stress was occurred; however, T/D was not induced; T/D group: T/D operated rats, received vehicle in two injections; T/D + CsA group: T/D-operated rats received two injections of 1 mg/kg CsA i.v; T/D + CsA group: T/D-operated rats received two injections of 5 mg/kg CsA i.v;T/D + Nort group: T/D-operated rats received two injections of 2 mg/kg Nort i.p; T/D + Nort group: T/D-operated rats received two injections of 10 mg/kg Nort i.p; T/D + CsA + Nort group: T/D-operated rats received two injections of CsA (1 mg/kg) and Nort (2 mg/kg), separately.

### Experimental T/D surgery

Before cervical dislocation, under a fully anesthetized condition with Ketamine HCl (80 mg/kg) and Xylazine (5 mg/kg), the depth of anesthesia was clinically monitored by *Toe Pinch Method* [25].

T/D surgery was initiated by a vertical incision in the scrotal zone. Tunica albuginea was opened and the right testis was twisted 720 degrees in clockwise direction around spermatic cord. 1 h later, the testis was counter-rotated to the normal position and inserted into the scrotum. Then, the skin incision was suture and animals were kept in the cage until removing time.

After orchiectomy, decapitation was performed with anesthetic agents in all groups.

#### Short-term; Biochemical assays

For determination of oxidative stress in damaged tissue, biochemical tests were done following ipsilateral orchiectomy 4 h after detorsion. The samples were washed by very cold saline solution and quickly snap freezed at –80 ºC for assessment of MDA, SOD, CAT, GPx and caspase-3 levels by preparing supernatant. Briefly, after weighing, the tissue manually homogenized in 10% W/V with ice-cold Tris buffer (10 mM Tris, pH 7.4) for 3 min, and the produced solution was centrifuged at 4 °C for 20 min. at 12,000 rpm, and the gathered solution was used for biochemical analysis [26].

### Tissue MDA level

Peroxidation of polyunsaturated fatty acids in damaged cell membrane indicates the rate of oxidative stress in tissue [27–30]. Free MDA concentration, ultimate and reactive bi-products of lipid peroxidation was assayed using thiobarbituric acid reactive substance (TBARS), as described by Ohkawa et al*.* [31]. In brief, testes were homogenized in 1.15% KCl to make a 10% (w/v) homogenate. Then 0.9 ml of 1.8% sodium dodecyl sulfate, 1.5 ml acetic acid 20% (pH = 3.5) and 1.5 ml of aqueous TBA solution were regularly added to 0.1 ml of tissue homogenates. The prepared homogenates were centrifuged at 4,000 rpm for 10 min. The supernatant was applied to determination of MDA levels, spectrophotometrically (λ = 532 nm).

### SOD activity

SOD helps break down potentially harmful oxygen molecules in cells. This might prevent damage to tissues [32]. Using Paoletti and Mocali method, SOD was measured [33]. Briefly, tissue SOD activity was assayed based on its ability to inhibit NADH oxidation in reaction mixture and conversion of superoxide anions (O_2_^•−^) to H_2_O_2_ and molecular oxygen (O_2_). SOD activity was determined by a reduction in absorbance at 340 nm wavelength during the reaction.

### CAT activity

CAT activity in tissue was spectrophotometrically determined according to Aebi's method [34]. Tissue sections were homogenized in 1% triton X-100 and diluted with potassium phosphate as a buffer. The reaction was initiated following hydrogen peroxide (H_2_O_2_) addition, and CAT activity was quantified based on the ability of tissue CAT to decompensate H_2_O_2_ by calculating the decrease in absorbance at 240 nm wavelength.

### GPx activity

GPx activity was spectrophotometrically evaluated regarding the modified method of Paglia & Valentine [35]. The enzymatic reaction was initiated following H_2_O_2_ addition and the alteration in absorbance at 340 nm was applied to measure GPx activity using a spectrophotometer. GPx catalyzes the oxidation of glutathione (GSH), a reducing reagent, by reduction of H_2_O_2_ to H_2_O. This reaction is coupled to oxidation of NADPH (nicotinamide adenine dinucleotide phosphate, reduced form) to NADPH^+^.

### Caspase-3 level

Level of caspase-3 was measured by using ELISA detection kit based on the Biotin double antibody sandwich technology (Bioassay technology Laboratory kit, Catalog No. E0280Ra Yangpu District Shanghai, China). The colorimetric alteration of samples at 450 nm was applied to measure caspase-3 concentration (ng/ml) by drawing a standard [36].

### Mid -term; Histopathological analysis

#### Histological preparation

Histological alterations were studied by ipsilateral orchiectomy 24 h after detorsion, following a rapid cervical dislocation. The testicular specimens were individually immersed in Bouin’s fixative, dehydrated in alcohol, embedded in paraffin and were cut in 5 μm sections by a microtome (RM2235 Rotary Microtome). Tissue sections were deparaffinized and stained with hematoxylin and eosin (H&E). Histopathologic examination was performed by two experts at 200 × magnification with light microscopy under blindfold condition. To illustrate testicular histological injury, the 4-level grading scale of Cosentino score was used [36];

- Grade 1: normal structure with regular arrangement of germ cells;
- Grade 2: testicular injuries with less orderly, non-cohesive germ cells and closely packed seminiferous tubules;
- Grade 3: testicular injuries with disordered, sloughed germ cells with shrunken, pyknotic nuclei and less distinction in seminiferous-tubule borders;
- Grade 4: testicular injuries with coagulative germ cell necrosis and intensely packed seminiferous tubules.

Moreover, MSTD value for each specimen was assessed by measurement of 10 separate roundest seminiferous tubules using an optical microscope equipped with a micrometer.

### Evaluation of germ cell apoptosis using TUNEL assay

Germ cell apoptosis was evaluated by the TUNEL assay. The TUNEL method, which detects fragmentation of DNA in the nucleus during apoptotic cell death in situ, was employed using an apoptosis detection kit (TdT-FragelTM DNA Fragmentation Detection Kit, Cat. No. QIA33, Calbiochem, USA). All reagents listed below are from the kit and were prepared following the manufacturer's instructions. Five-μm-thick testis tissue sections were deparaffinized in xylene and rehydrated through a graded ethanol series as described previously. They were then incubated with 20 mg/ml proteinase K for 20 min and rinsed in TBS. Endogenous peroxidase activity was inhibited by incubation with 3% hydrogen peroxide. Sections were then incubated with equilibration buffer for 10–30 min and then TdT-enzyme, in a humidified atmosphere at 37 °C, for 90 min. They were subsequently put into pre-warmed working strength stop/wash buffer at room temperature for 10 min and incubated with blocking buffer for 30 min. Each step was separated by thorough washes in TBS. Labelling was revealed using DAB, and sections were dehydrated, cleared and mounted [3, 37, 38].  The positive staining of TUNEL cell numbers was scored in a semiquantitative manner in order to determine the differences between the control group and the experimental groups in testes tissue. The numbers of the positive staining were recorded as absence ( −), a few ( ±), few ( +), medium (+ +), high (+ + +) and very high (+ +  + +). This analysis was performed in at least eight areas per testis section, in two sections from each animal at × 200 magnification under light microscopy by two experts who were unaware of the study design (Fig. 3).

#### Long-term; Semen Analysis

Testicular I/R and the effects of administrated drugs on semen parameters were evaluated 30 days after operation. The epididymal sperm concentration was measured by Yokoi et al*.* method [39]. Briefly, epididymis completely was cut, squashed by tweezers in physiologic saline and incubated at room temperature for 5 min to allow the egress of spermatozoa from epididymal to fluid. Then, supernatant fluid was diluted 1/100 with a solution containing 5 g sodium bicarbonate, 1 ml formalin (35%), and for sperm dying was added 25 mg eosin per 100 ml of water. The mixture was centrifuged at 6,000 rpm for 20 min at room temperature. Live sperms remained unstained following staining; whereas, those that seemed any pink or red coloration were classified as dead [3]. Then 10 μl of the diluted sperm suspension was transferred to each counting chamber of hemocytometer and the number of alive sperms was counted with a light microscope at the magnification of 200 × . Using a standard method which possesses a score ranging from 0 to 100%, the progressive sperm motility percentage was visually recorded under light microscopy (400 × magnification). Final motility score estimations calculated by mean value from 4 different fields in each sample.

### Statistical analysis

Statistical analysis was conducted by GraphPad Prism version 8.00. The results were reported as mean ± SD. Shapiro–Wilk test was used for evaluation of the normal distribution of numerical variables. Comparisons between the groups were assessed by one-way analysis of variance for variables with normal distribution, and in case of differences, multiple comparisons were conducted using the Tukey test. For the non-normal distribution variables, comparisons between groups were evaluated by Kruskal–Wallis analysis; if there was a difference, multiple comparisons were made using the Mann Whitney U test. Statistical significance level was set at *p* ≤ 0.05.

## Results

### Biochemical analyses findings

The level of testicular MDA and caspase-3 and GPx, CAT and SOD activities in all experimental groups are reported in Table 1. According to the observed results significant statistical differences were detected in the level of antioxidant enzymes between the T/D and control groups (*p* < 0.001). Protective effects in both pre- and post-treatment with Nort and CsA in selected doses, were observed by a reduction in lipid peroxidation after testicular I/R in rats. The levels of MDA in testis tissue of CsA, Nort and CsA + Nort double injected animals were lower than T/D group. The SOD, GPx and CAT activities in the treatment groups were enhanced in compared to the T/D group. Our results showed that CsA, Nort and CsA + Nort could not thoroughly decrease activity of the caspase-3, but treatment in all groups subsided testis tissue injuries; this amelioration was statistically significant between all treated groups and T/D group (*p* < 0.001). CsA + Nort treated group, indicated no significant difference in results with groups which treated by high dose of CsA and Nort.

**Table 1:** Levels of MDA and CAT, SOD, GPx and caspase-3 activities 4 h after testicular detorsion

Group	MDA (nmol/g wet tissue)	CAT (IU/g wet tissue)	SOD (IU/g wet tissue)	GPx (IU/g wet tissue)	caspase-3 (ng/ml)
**Control**	116.09 ± 5.43***	354.19 ± 9.64***	1955.23 ± 10.70***	706.53 ± 32.26***	0.275 ± 0.024***
**T/D**	183.02 ± 18.26	264.71 ± 19.53	1581.13 ± 45.01	562.63 ± 31.99	0.523 ± 0.016
**CsA 1**	155.03 ± 10.08***	276.35 ± 10.28	1722.82 ± 41.54***	615.16 ± 15.44*	0.425 ± 0.024***
**CsA 5**	133.45 ± 5.27***,†	300.71 ± 13.51*,†	1752.49 ± 43.73***	630.20 ± 25.31***	0.363 ± .028***,^φφφ^
**Nort 2**	162.50 ± 9.24***	267.24 ± 9.09	1681.14 ± 17.01***	604.49 ± 8.38*	0.430 ± 0.037***
**Nort 10**	147.54 ± 5.27***,†	290.71 ± 12.58*,†	1736.49 ± 45.78***	625.10 ± 14.48***	0.342 ± .028***,^φφφ^
**CsA 1 + Nort 2**	158.30 ± 2.46***	281.20 ± 7.50*,†	1758.89 ± 44.87***	625.40 ± 21.32***	0.330 ± .029***

^*^p < 0.05 compared with T/D group

^***^ *P* < 0.001 compared with T/D group

^†^ *P* < 0.05 compared with CsA1mg/kg

^φφφ^ *P* < 0.001 compared with 1 mg/kg

(*n* = 6)

### Histological analyses

**Fig. 2:**
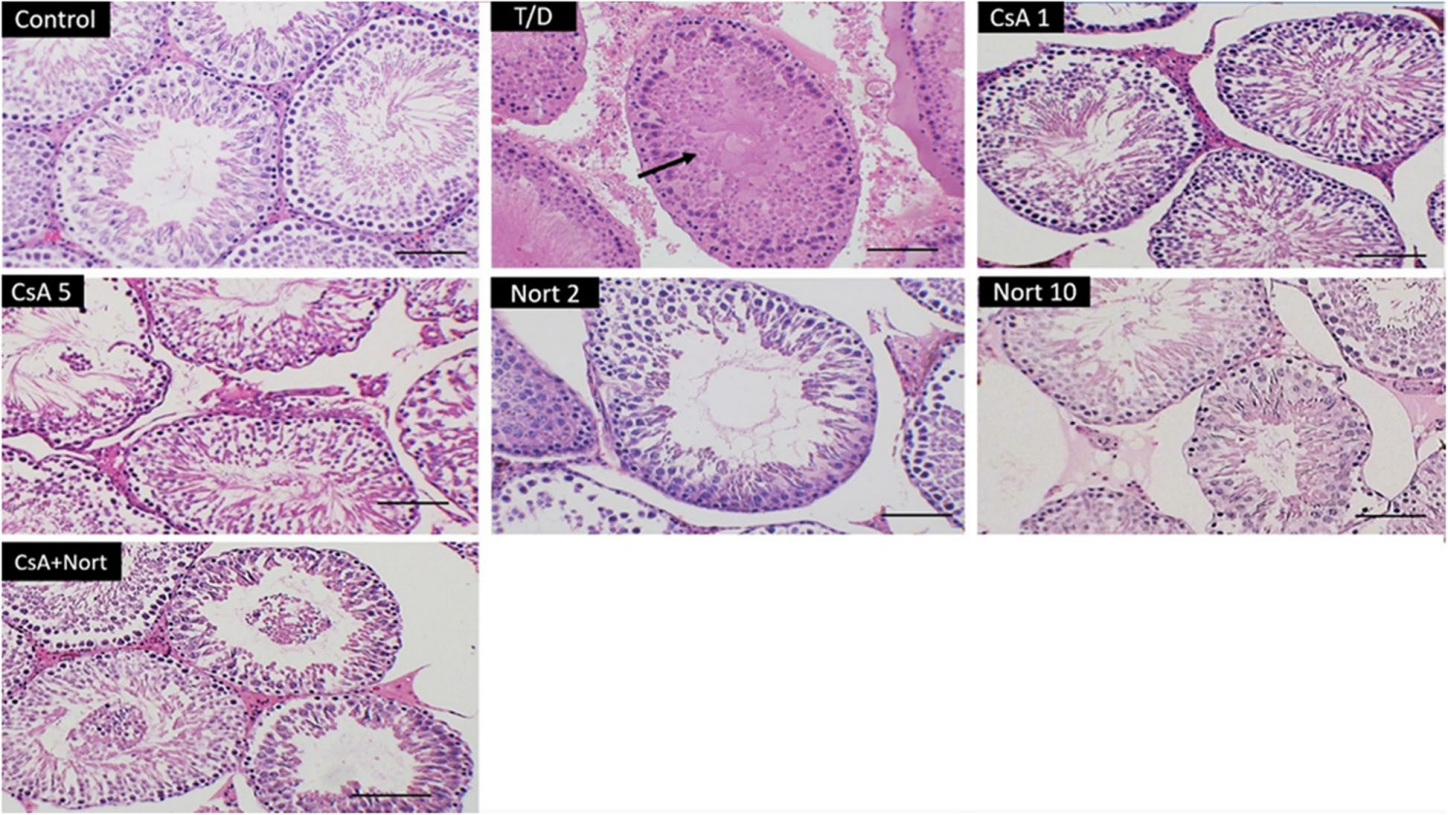
Histological appearances in ipsilateral testes groups: Control, T/D, CsA 1, CsA 5, Nort 2, Nort 10 and CsA + Nort (1 and 2 mg/kg). The ischemic alterations and coagulative necrosis (black arrow) were observed, and the orderly arrangement of germ cells was impaired in T/D group. After treatment with CsA, Nort, and CsA + Nort spermatogenesis was restarted and orderly structure of germ cells with a few mature spermatids was observed within seminiferous tubules (H&E; Scale bar: 50, magnification × 200)

Table 2 indicates the histological parameters in the all groups. In the control animals a normal thickness in cell layers a normal morphology in seminiferous tubules with intact germinal epithelium were observed. Pathological signs were observed in testis 24 h after fixation in natural position in T/D rats. After I/R, we observed a comprehensive distortion of tubules with a significant reduce in MSTD (Fig. 2). Testicular I/R affected the spermatogenesis in the T/D group, consequently, a reduction in the mean testicular scores were observed. Except Nort2 group, spermatogenesis parameters significantly enhanced between T/D and treated groups (*p* < 0.001), and these results were significant between CsA1 and 5 mg/kg (*p* < 0.001) and between CsA1 and CsA + Nort (*p* < 0.01). Treatment with selected agents remarkably improved Cosentino’s score in all of treated groups 24 h after detorsion.

**Table 2:** Histological evaluation by using MSTD values and Cosentino’s scores 24 h after detorsion

Group	MSTD (µm)	Grade
**Control**	295.7 ± 11.72***	1
**T/D**	213.0 ± 13.67	3
**CsA 1**	253.2 ± 17.80***	2
**CsA 5**	269.2 ± 18.05***, †††	2
**Nort 2**	225.8 ± 6.73	2
**Nort 10**	245.8 ± 10.61***,†	2
**CsA 1 + Nort 2**	259.7 ± 7.94***, ††	2

^***^^p < 0.001 compared with T/D group^

^†††^ *P* < 0.001 compared with CsA 1 mg/kg

^††^ *P* < 0.01 compared with CsA 1 mg/kg

^†^ *P* < 0.05 compared with Nort 2 mg/kg

Cosentino’s score: 1. Minimal or no evidence of injury, 2. Slight injury, 3. Mild injury, 4. Moderate injury

(*n* = 6)

### Immunohistochemical analyses findings

**Fig. 3:**
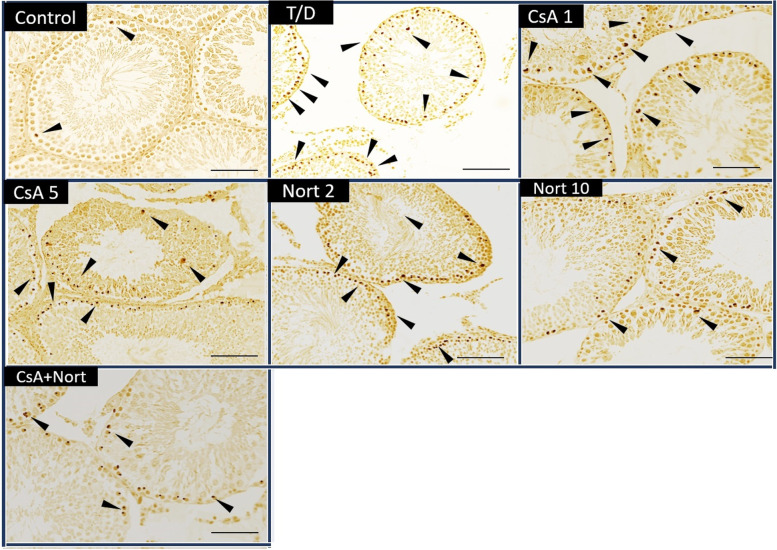
Apoptotic nuclei and seminiferous tubules. A few apoptotic nuclei were observed in Control group. Apoptotic germ cells significantly increased following T/D. After treatment with CsA 5, Nort 10 and CsA 1 + Nort 2, the number of germ cell apoptosis (arrowhead) was reduced compared with T/D group (Scale bar: 50 µm. magnification × 200)

Figure 3 shows the results of DNA laddering semi-qualitative evaluation. By conducting double labeling, the TUNEL positive nuclei and surrounding normal nuclei were compared with each other, and then we evaluated the anatomical structures. In this regard only the cells with fulfilled morphological criteria were considered as TUNEL-positive cells. The number of germ cell apoptosis was semi-quantitatively higher in T/D group than control group. Treatment with CsA at 5, Nort at 10 mg/kg and CsA 1 + Nort 2 dosages markedly reduced the reactivity and the number of germ cell apoptosis (Table 3; Fig. 3).

**Table 3:** Semiquantitative comparison of the positive staining of TUNEL cell numbers in testicular tissues for each group

	**Control**	**T/D**	**CsA 1**	**CsA 5**	**Nort 2**	**Nort 10**	**CsA + Nort**
**TUNEL**	±	+ + + +	+ + + +	+ +	+ + + +	+ +	+ +

The numbers of the positive staining were recorded as a few ( ±), few ( +), medium (+ +), high (+ + +) and very high (+ +  + +) (n: 6 for each group)

### Semen analysis findings

**Fig. 4:**
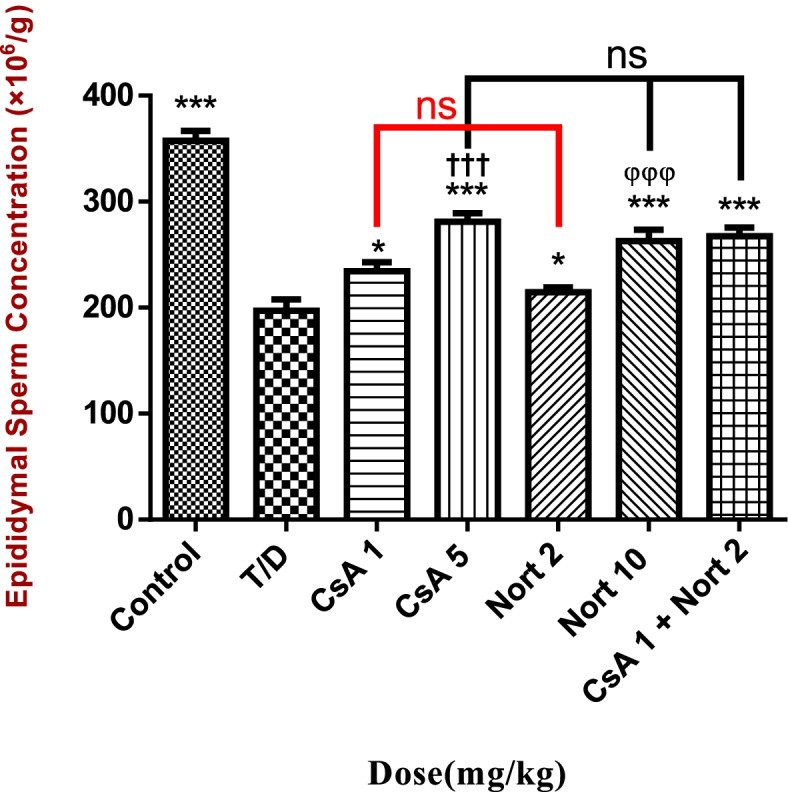
Epididymal sperm concentration. *p < 0.05 compared with T/D group. ***p < 0.001 compared with T/D group. †††*P* < 0.001 compared with group CsA 1 mg/Kg. φφφ*P* < 0.001 compared with group Nort 2 mg/kg. (*n* = 4)

The long-term effects of testicular T/D on epididymal sperm concentration and motility were noxious. Double injection of CsA (1and 5 mg/kg) dose-dependently improved the reduced sperm concentration in compared with T/D group (*p* < 0.05 and *p* < 0.01, respectively) (Fig. 4).

**Fig. 5:**
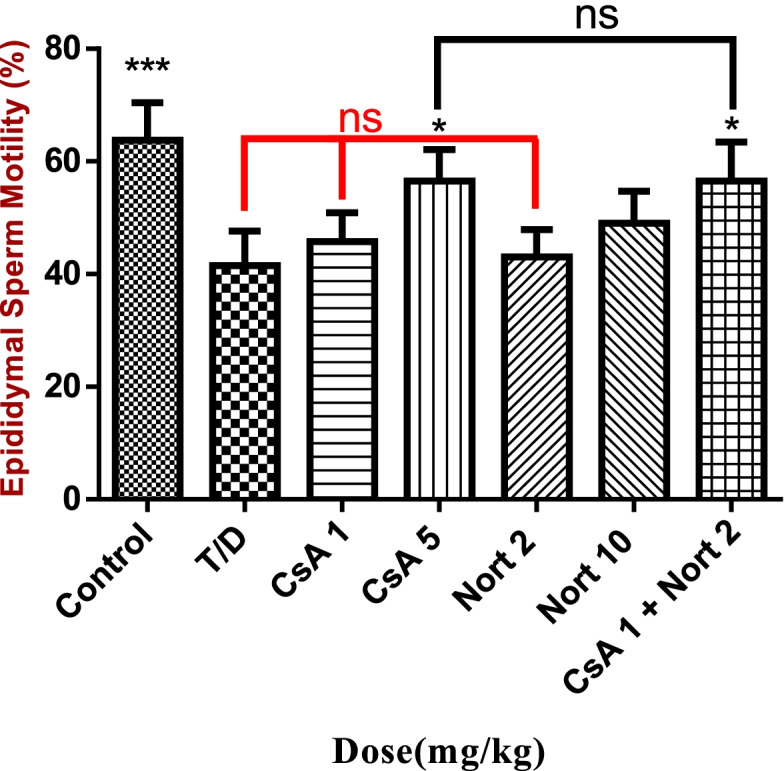
Epididymal sperm motility.***P < 0.001 compared with T/D group. *P < 0.05 compared with T/D group. (*n* = 4)

Reduced percentage of sperm motility resulted from testicular T/D did not improve by CsA 1, Nort 2 and Nort 10 mg/kg. This value significantly improved at 5 mg/kg and CsA1 + Nort 2 dosages compared with T/D group (*p* < 0.05) and CsA + Nort treated group, showed no significant difference in results with CsA 5 treated group (Fig. 5).

## Discussion

In this study, the antioxidant, anti-apoptotic and epididymal spermatic effects of injected CsA, Nort and their extemporaneously administration in selected doses were investigated on testicular T/D damages. Based on the obtained results, an equal or even better efficacy was obtained in simultaneous administration of low-dose of CsA and Nort combination than either drug alone at higher doses.

Rotation of testis around the axis of spermatic cord and cutting off the blood supply to the testicle is an acute pathologic status and rapid diagnosis and correct medical treatment should be pursued promptly to prevent progressive testes injuries [40]. Although restoration of blood flow by surgical detorsion is unequivocally essential, studies have been proven that it may induce a cascade of pathological reactions which can accelerate tissue ailment [41].

Related adverse effects of tissue reperfusion are the activation of immunity and inflammatory mediators by transmigration of neutrophils from endothelium into testis interstitium and recruitment of inflammatory cytokines lead to apoptosis motivation [42, 43]. Indeed, neutrophils-induced pro-inflammatory cytokines like TNF-α, IL-1β, IL‐2 in reperfused tissue stimulate nuclear transcription factor (NF-κB) activation which causes apoptosis and germ cell mass attenuation [2, 8]. On the other hand, overproduction of ROS can disrupt mitochondria *via* mPTP directly, or facilitate DNA cleavage in germ cells through the caspase activation indirectly, which can intensify germ cell-specific apoptosis in damaged tissue [22]. So, prevention of mPTP opening in I/R is a compensatory mechanism to reduce the effects of insults [44]. Literatures have been expressed the protective role of immunophilin ligands, especially CsA by mPTP suppression in some organelles such as brain, heart, liver and testis following I/R [13, 16, 45, 46].

To prevent of testes malfunction, studies suggest that treatment with immunosuppressive, anti-apoptotic and anti-inflammatory agents can protect the tissue in T/D [24, 43, 46, 47]. In line these information, CsA as a immunosuppressant can implicate in control of these processes positively; both mPTP-dependent which is a key regulator of cellular energy depletion to stress-induced apoptosis, and mPTP-independent mechanisms by inhibiting calcineurin pathway activation [37, 38]. mPTP with proteinaceous structure predominantly forms by integration of three proteins named VDAC-ANT-CypD with an inorganic phosphate carrier [48]. Binding these component in oxidative stress conditions is crucial for opening of pore to evacuate mitochondrial over-loaded Ca^+2^ [6]. Cyclophilin D (CypD), one of mitochondrial cyclophilins, is mPTP activator, which located on the inner membrane and has regulatory role in protein folding and/or conformational change in proteins of pore [48].

CsA is a ligand for cyclophilins and its linkage by cyclophilins produces two downstream; binding to cyclophilins A (CypA) results in inhibition of calcineurin pathway and binding to CypD which results to mPTP inhibition. Immunosuppressive effects are distinctly performed by calcineurin pathway, while cytoprotective effects in I/R conditions are mediated by blocking of CypD and keeping mPTP in closed form [37, 48].

CypA is a protein from the immunophilin family and binding of CypA to CsA to induce the immunosuppressive ability through formation a complex between CsA and CypA to stop the calcineurin activity. Calcineurin triggers NF-κB activation by Ca^+2^/calmodulin-dependent protein phosphatase [38]. Calcium-dependent signal transduction is an essential factor for the induction of cytokine expression by stimuli in leukocytes. The subsequent of inhibited calcineurin-catalyzed dephosphorylation is the reduced production of TNF-α and IL-2 [47, 49].

CsA can directly inhibit mPTP by one of two ways; First, CsA is a reasonable size and can inhibit movement of large solutes influx [48]. Secondly, CsA induces inhibitory conformational changes in CypD conformation by preventing CypD binding to the PiC/ANT strand and keeps pore in closed form [6]. CsA protects cells against ΔΨm depolarization and moderates oxidative stress induced apoptosis *via* ROS generation in cardiomyocytes [45]. For a complex and multifaceted condition such as TBI and acute brain injury and diffuse axonal injury models which is related to mPTP inhibition by binding to CypD, CsA exhibited neuroprotective potency [18]. In myocardial I/R, CsA preserves mitochondrial morphology independent of calcineurin inhibition [15]. Administration of CsA in heart failure was associated with a greater post-ischemic recovery and smaller infarction size by pore closing [15, 50].

Beside these, a previous study demonstrated the beneficial effects of concomitant use of CsA with prednisolone and melatonin in reducing of contralateral testicular damage after ipsilateral testicular T/D by immunological mechanism [37, 38, 44, 46].

Similar to CsA, recently reported that treatment with Nort can reduce lipid peroxidation, oxidative stress and germinal cell apoptosis in a experimental model of testicular T/D injury [2]. Various evidences indicate that treatment with nortriptyline can attenuate transmigration of neutrophils from endothelium and preclude endothelium–monocyte adhesion stimulated by TNF-α in I/R conditions [21, 51]. In calcium overload status, Nort increases the Ca2 + retention capacity of mitochondria, delays Ca2 + induced Ca2 + release, exerts significant resistance to calcium overload-induced injury and inhibits loss of mitochondrial membrane potential. Nort exerts notable resistance to calcium overload-induced injury, inhibition of arachidonic acid release and lipid peroxidation which attributes to prevention of mPTP assembling in short time and inhibits the collapse of mitochondrial membrane potential and mPTP-mediated release of apoptogenic factors such as AIF from mitochondria [19, 21, 51]. Nort reduces oxidative stress factors such as elevated H_2_O_2_, superoxide, lactate dehydrogenase generation in ischemied/reperfused cells which are the onset of acute inflammatory responses, and augments intracellular antioxidant enzymes and helps cell viability in hypoxia/normoxia situations [51].

In the current study we observed a decline in caspase-3 activity and MDA level Furthermore an elevation of GPx and SOD activities in testis tissue of treated animals with CsA and Nort 4 h after detorsion were reported. However, the lowest MDA level after T/D were detected in combined treatment. So, combination CsA and Nort significantly augmented ameliorative properties compared with mono-therapy in I/R injury. In the histopathological evaluation, as a result of exerted testicular I/R, the apparent deterioration and irregularity in the interstitial and seminiferous tubules structures and the number of cells in the spermatogenic series, were observed. As reported in previous studies, treatment by CsA and Nort diminished testicular dysfunction and histopathological abnormalities after I/R in mid-term and long-term and findings in injured tissue indicated significant improvement in histological and spermatic criteria in treated groups after I/R lesion. However, the most profound effect was observed with their combinatorial treated group. These results identified that CsA and Nort play a significant cytoprotective role in attenuating T/D injury by alleviating cell damages in extemporaneously administrated group. So, our findings clarify the mPTP-induced illnesses such as T/D can be healed by mPTP blockers and it could be hypothesized that the concurrent therapy may produce better protective effects in I/R insults. Although, this combination therapy may represent a promising outcome against T/D injury, additional researches are inevitable to investigate the perfect mechanisms in T/D and its well-known side effects must be considered as a restrictions on clinical use [52].

## Conclusion

Our findings could illustrate evidences about the protection against oxidative damage and apoptosis in T/D by co- treatment with CsA and Nort in accordance mPTP involvement.

The combination therapy at low doses indicated comparable or even better effects than each drug alone in higher dose and could demonstrated that current therapy reinforces the potency of treatment. As seems probable, this method might provide an innovative and safety strategy to develop a satisfactory way to reduce side effects of administrated drugs and an effective management with adjuvant therapy during T/D surgery.

## Data Availability

The datasets generated during and/or analyzed during the current study are not publicly available due to confidential issues but are available from the corresponding author on reasonable request.
